# Terahertz Spectroscopy and Density Functional Theory for Non-Destructive Analysis of Anticoagulant Warfarin

**DOI:** 10.3390/molecules30081791

**Published:** 2025-04-16

**Authors:** Jiawei Li, Cong Zhang, Xiaohui Wang, Jinjing Zhang, Hanwen Liu, Xu Wu

**Affiliations:** 1 Shanghai Key Laboratory of Modern Optical System, Terahertz Technology Innovation Research Institute, School of Optical-Electrical and Computer Engineering, University of Shanghai for Science and Technology, Shanghai 200093, China; 223330868@st.usst.edu.cn (J.L.); 232200325@st.usst.edu.cn (J.Z.); 243350755@st.usst.edu.cn (H.L.); 2 Laboratory of Chemical Biology, Changchun Institute of Applied Chemistry, Chinese Academy of Sciences, Changchun 130022, China; cong.zhang@ciac.ac.cn (C.Z.); xiaohui.wang@ciac.ac.cn (X.W.); 3 School of Applied Chemistry and Engineering, University of Science and Technology of China, Hefei 230026, China; 4 Shanghai Institute of Intelligent Science and Technology, Tongji University, Shanghai 200092, China; 5 Hainan Institute, East China Normal University, Sanya 572025, China

**Keywords:** terahertz spectroscopy, DFT calculation, warfarin, pharmaceutical quality control, qualitative identification, quantitative detection

## Abstract

Pharmaceutical quality control plays a critical role in safeguarding patient safety and ensuring therapeutic efficacy. However, conventional analytical methods are often hindered by laborious procedures and complex chemical preparation requirements. This study presents a rapid, non-destructive pharmaceutical analysis approach by introducing terahertz spectroscopy for the dual-parametric detection of the anticoagulant warfarin. Characteristic absorption peaks of warfarin within the 4–10 THz range were experimentally identified and theoretically resolved through density functional theory calculations, employing both single-molecule and unit cell models. Furthermore, three strong absorption peaks were selected to construct multivariate regression models correlating spectral parameters (peak intensity and area) with warfarin weight, achieving a detection limit of 0.641 mg within a 5 min analytical workflow. This approach enables simultaneous molecular fingerprint identification and quantitative determination without chemical modification, meeting the requirements for the rapid screening of active pharmaceutical ingredients.

## 1. Introduction

The relationship between drug quality and human health manifests in dual dimensions. Pharmaceuticals containing standardized active pharmaceutical ingredients (APIs) constitute lifesaving therapeutic agents, whereas counterfeit drugs with unqualified APIs present significant public health risks through therapeutic failure and toxicity. Beyond endangering patient safety, these counterfeit medicines adversely affect pharmaceutical brand equity, compromise governmental regulatory credibility, and distort global market dynamics [1,2,3]. It has been reported that counterfeit medicines represent nearly 1% of pharmaceutical market in developed countries but escalate to 30–60% prevalence in underdeveloped regions, including Latin America, Southeast Asia, and sub-Saharan Africa [4]. WHO estimates associate counterfeit medicines with approximately 1 million annual fatalities, including 200,000 fatalities in Africa alone [5,6]. It is necessary to develop pharmaceutical detection technology to safeguard patient welfare.

Currently pharmaceutical analytical methods include high-performance liquid chromatography (HPLC) [7,8], mass spectrometry (MS) [9,10], and Raman spectroscopy (RS) [11,12]. HPLC technology achieves compound separation through high-pressure mobile phase transport coupled with stationary phase interactions, offering high sensitivity and quantitative precision. However, its utility is constrained by prolonged analysis cycles (typically 20–30 min/sample), expensive column consumables, and labor-intensive sample preparation [13]. MS technology is based on m/z (mass/charge)-dependent ion separation in electromagnetic fields, providing high sensitivity and excellent structural elucidation capacity. However, it requires complex instrumentation and substantial operating costs [14]. RS is based on the inelastic photon scattering and characterizes molecular vibrational fingerprints, enabling rapid analysis. However, it suffers from potential photothermal sample degradation [15]. Consequently, there is an urgent need for a rapid, accurate, economical, and non-destructive method for pharmaceutical detection.

Terahertz (THz) waves, occupying 0.1–10 THz in the electromagnetic spectrum between microwaves and infrared waves [16,17,18], exhibit three unique properties critical for pharmaceutical analysis: (1) Their non-ionizing nature with photon energies (0.4–41 meV), insufficient for molecular ionization, ensures non-destructive sample interrogation [19]. (2) Their molecular fingerprint specificity arises from resonant interactions involving intra-molecular vibration, inter-molecular vibrations, and crystalline lattice modes. THz spectroscopy presents significant advantages for drug detection due to the vibrations, rotations, and weak interactions of most molecular groups that fall within the frequency range of THz radiation [20,21,22]. (3) Their capability to penetrate through dielectric pharmaceutical packaging materials, including plastics, polymers, and cellulose matrices, enables non-invasive APIs detection [23]. These properties collectively establish THz spectroscopy as a unique approach for pharmaceutical detection, offering crystallographic discrimination, non-destructive analysis, and simple sample preparation.

Recent advancements demonstrate the capabilities of THz spectroscopy in pharmaceutical characterization. In 2017, Yuan et al. achieved norfloxacin quantification via a dual peak at 0.2–1.7 THz [24]. In 2018, Uroš Puc et al. investigated the absorption characteristics of melatonin at 1.5–4.5 THz [25]. In 2020, Yin et al. resolved structural analogs among flavonoids, including baicalein, baicalin, apigenin, quercetin, naringenin, hesperetin, daidzein, genistein, puerarin, and gastrodin, based on their THz spectra at 0.2–2.5 THz [26]. In 2023, Xu et al. measured the absorption and refraction spectra of compound banlangen tablets at 1.10–1.17 THz, and further studied differences in both absorption and refractive index spectra across various thicknesses, batches, and manufacturers [27]. THz technology has been extensively studied in pharmaceutical analysis, but the spectral properties of warfarin remain insufficiently explored despite its widespread clinical use [28,29,30]. While Wu et al. investigated the absorption characteristics of warfarin sodium in the 10–16 THz range, the sub-10 THz range, which is critical for the pharmacologically relevant crystalline phase of warfarin, remains unexplored [31].

In this study, we present a dual-parametric THz spectroscopic strategy for the widely used anticoagulant warfarin. Firstly, the absorption peaks of warfarin within the frequency range of 4 to 10 THz were identified. Subsequently, these peaks were theoretically validated through the density functional theory (DFT) method, incorporating both molecular and crystalline configurations. The experimental and theoretical THz spectrum enabled the non-destructive qualitative identification for warfarin with a crystalline phase resolution. Furthermore, we developed a multivariate quantification model correlating peak parameters (peak intensity, integrated peak area) with warfarin mass. This THz spectral approach eliminated chemical derivatization while reducing analysis time from hours to minutes compared to HPLC.

## 2. Results and Discussion

### 2.1. Experimental THz Spectral Characterization of Warfarin

**Figure 1:**
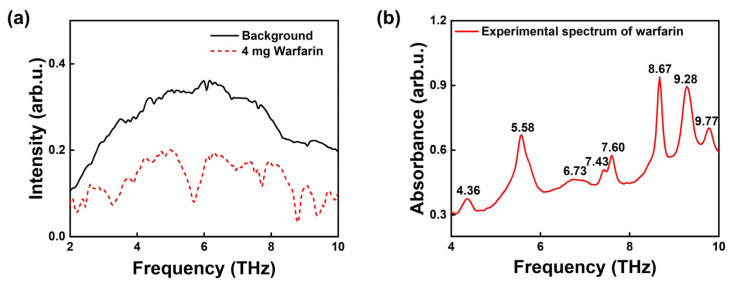
THz absorption spectra of warfarin in 4 to 10 THz. (**a**) frequency spectrum; (**b**) absorption spectrum.

Figure 1a demonstrates appropriate experimental conditions through signal intensity comparison, where the 4 mg warfarin sample signal (0.2 arb.u.) falls between 1/2 and 2/3 of the background maximum (0.36 arb.u.), confirming optimal sample thickness and mass. The corresponding absorption spectrum is shown in Figure 1b. It reveals that warfarin exhibits eight characteristic absorption peaks at 4.36, 5.58, 6.73, 7.43, 7.60, 8.67, 9.28, and 9.77 THz within the 4–10 THz range. At 6.73 THz, a series of absorption peaks were observed, resulting from the superposition of multiple crystal cell vibrational modes. We identified and labeled the most prominent absorption peaks among them.

### 2.2. THz Vibrational Modes Analysis of Warfarin

**Figure 2:**
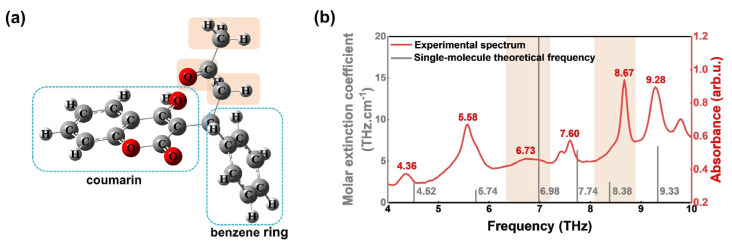
Simulated results for the single molecule of warfarin. (**a**) Single-molecule modeling; (**b**) comparison of theoretical and experimental frequency for the single molecule.

The warfarin molecular structure, as illustrated in Figure 2a, comprises a coumarin core (C_9_H_5_O_3_), a benzene ring (Ph), a methylene group (CH_2_), a methyl group (CH_3_), and a ketone group. The DFT calculation results of a single warfarin molecule are shown in Figure 2b. This computational protocol has been successfully validated against experimental data for diverse compounds such as serotonin [32], 2-thiobarbituric acid [33], and allantoin [34]. These studies show that the DFT-D3 correction method accurately predicts the low-frequency vibrational modes of molecular crystals, with deviations between the calculated and measured peaks typically within 0.2 THz. This provides a reliable theoretical validation for the calculation method used in our study. Due to the limitations of the computational method, particularly the neglect of inter-unit cell interactions under periodic boundary conditions, the two experimental peaks at 7.43 and 9.77 THz were not observed in the calculation results. Therefore, we focused our discussion on the remaining six peaks. The theoretical predicted six vibrational peaks within the 4–10 THz range, located at 4.52 THz, 5.74 THz, 6.98 THz, 7.74 THz, 8.38 THz, and 9.33 THz, respectively.

Theoretical calculations demonstrated strong agreement for four characteristic peaks (4.36, 5.58, 7.60, and 9.28 THz), with deviations of ≤0.16 THz, indicating their origin in the intramolecular vibrations of isolated warfarin molecules. As demonstrated by Grimme et al. [35], the D3 correction reduces the computational error of low-frequency vibrations in molecular crystals, ranging from 0.5 THz to 0.2 THz. Based on this, we adopted a threshold of ≤0.16 THz for strong agreement, while deviations exceeding 0.2 THz were classified as significant discrepancies. However, significant discrepancies emerged for the 6.73 THz (Δ = 0.25 THz) and 8.67 THz (Δ = 0.29 THz) peaks, necessitating the consideration of crystalline lattice interactions to account for these vibration modes. As shown in Table 1, the four characteristic peaks predicted by DFT exhibit a consistent redshift compared to the experimental data. Possible reasons include the neglect of anharmonic effects in the harmonic approximation, limitations of the basis set in describing weak intermolecular interactions, and the tendency of the B3LYP-D3 method to overestimate bond stiffness. Although empirical scaling factors (~0.97) could partially correct this effect [36], we intentionally present unscaled data to maintain methodological transparency and avoid introducing additional empirical parameters.

**Figure 3:**
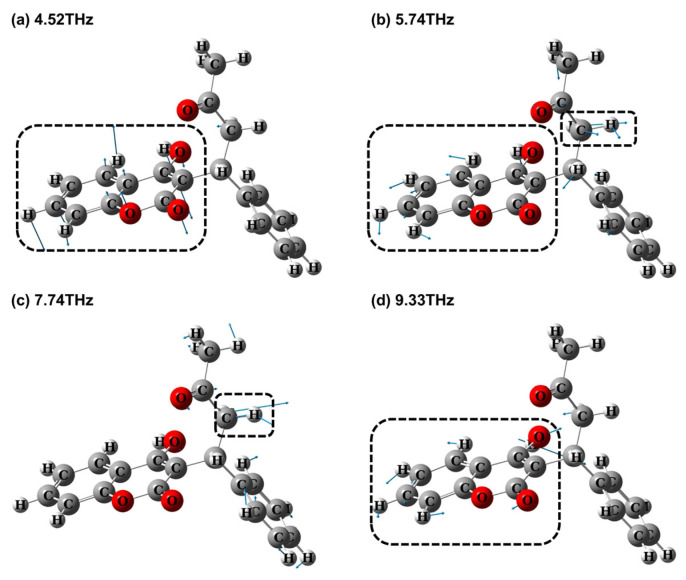
Theoretical vibration modes of warfarin at different theoretical peaks. The arrows show the direction of vibration, and the length of the arrow is the intensity of vibration. (**a**) 4.52 THz; (**b**) 5.74 THz; (**c**) 7.74 THz; (**d**) 9.33 THz.

To understand the vibrational modes of the observed THz absorption peaks, we first analyze the four peaks (4.36, 5.58, 7.60, and 9.28 THz) that align well with the single-molecule model results. For each absorption peak identified through the DFT calculations, the corresponding molecular vibration mode is visualized in Figure 3. Specifically, the theoretical peak at 4.52 THz corresponds well with the measured peak at 4.36 THz, which is attributed to the out-of-plane bending vibration of the coumarin core (C_9_H_5_O_3_), as illustrated in Figure 3a. The theoretical peak at 5.74 THz aligns closely with the measured peak at 5.58 THz, which is primarily attributed to both rocking vibrations of the coumarin core (C_9_H_5_O_3_) and benzene ring (Ph), as depicted in Figure 3b. The theoretical peak at 7.74 THz corresponded to the measured peak at 7.60 THz, which mainly originates from the rocking vibration of CH_2_ and the rocking vibration of Ph, as shown in Figure 3c. The theoretical peak at 9.33 THz corresponded to the measured peak at 9.28 THz, which was mainly generated by the in-plane bending vibration of C_9_H_5_O_3_, as shown in Figure 3d.

**Figure 4:**
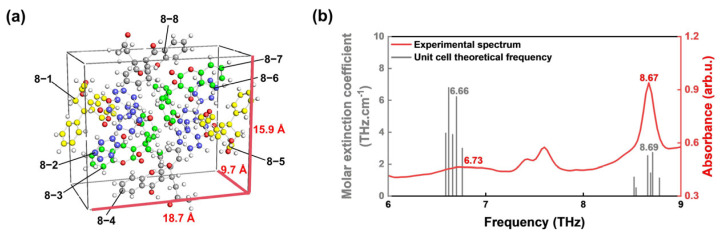
Simulated results for the warfarin unit cell. (**a**) Unit cell modeling. White atoms represent hydrogen (H), red atoms correspond to oxygen (O), while yellow, blue, green, and gray atoms denote carbon (C) from the eight warfarin monomers, with each color representing a symmetric pair. (**b**) Comparison of theoretical and experimental peak positions for the unit cell.

To further investigate the influence of intermolecular interactions for 6.73 THz and 8.67 THz peaks, we extended our theoretical calculations to the warfarin unit cell. The unit cell structure, comprising eight warfarin monomers (labeled 8-1 to 8-8), is depicted in Figure 4a. The unit cell dimensions measure 18.7 Å in length, 9.7 Å in width, and 15.9 Å in height. Theoretical predictions are compared with experimental results in Figure 4b. Experimentally observed peaks at 6.73 THz arise from the collective contribution of five resonant modes, corresponding to the coupled vibrations of warfarin monomers 8-1, 8-3, 8-4, 8-5, and 8-6. Compared to single-molecule calculations, the results of unit cell calculations show significant differences. This discrepancy arises because single-molecule calculations do not account for intermolecular interactions, such as hydrogen bonding. These interactions lead to notable peak broadening and a reduction in absorption intensity [37]. In contrast, the peak at 8.67 THz originates from six overlapping resonant modes, with all eight warfarin monomers in the unit cell participating in the vibrational coupling. Incorporating intermolecular interactions into the unit cell calculations improved the agreement with experimental data for the peaks at 6.73 and 8.67 THz, reducing the deviations to within 0.07 THz. This enhanced correlation between theory and experiment underscores the importance of intermolecular interactions in determining the vibrational properties of warfarin and validates the reliability of our theoretical approach in predicting its spectroscopic characteristics.

### 2.3. Quantitative Analysis of Warfarin Based on Dual THz Parameters

**Figure 5:**
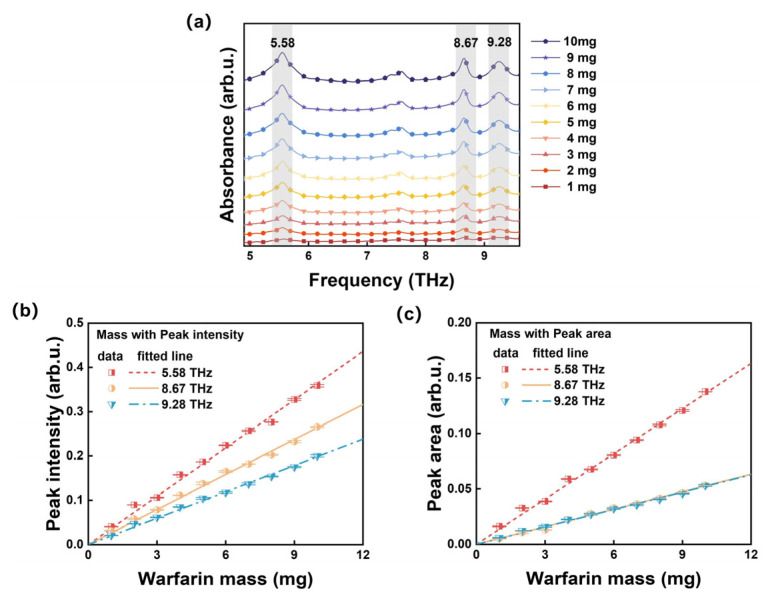
THz absorption spectra and quantitative analysis of warfarin–COC tablets. (**a**) THz absorption spectra of warfarin–COC tablets with different warfarin masses; (**b**) mass versus peak intensity; (**c**) mass versus peak area.

Based on the absorption characteristic peaks of warfarin in 4–10 THz, the quantitative analysis of warfarin mass is further outlined in this Section. Warfarin–COC mixed samples were analyzed using THz spectrometer. The three strongest absorption peaks (5.58, 8.67, and 9.28 THz) were selected for quantitative evaluation. The integration ranges for these peaks were carefully chosen based on the peak shape and baseline characteristics. For each peak, the integration range was defined from the point where the signal significantly deviates from the baseline to the point where the signal returns to the baseline. Specifically, the integration range for the 5.58 THz peak was 5.11–6 THz; for the 8.67 THz peak, it was 8.46–8.86 THz; and for the 9.28 THz peak, it was 8.92–9.53 THz. This approach ensured accurate peak area quantification while avoiding interference from surrounding noise or overlapping signals. As shown in Figure 5, the results demonstrate clear and consistent increase in both peak intensity and area as the warfarin mass increased from 1 mg to 10 mg. Based on the data in Figure 5a, we quantified warfarin mass by investigating the relationship between mass and THz spectral characteristics. The spectra were baseline corrected and dispersed to better examine the spectral characteristic changes at various masses. Specifically, we analyzed the changes in both peak intensity and integrated area of the three strongest peaks as functions of warfarin mass.

The linear relationship between peak intensity and warfarin mass is depicted in Figure 5b, where we constrained the intercept of the fitting function to zero for improved accuracy. The corresponding linear fitting function, which models the mass (*x*, in mg) versus peak absorption intensity (*y*, in arbitrary units), is shown in Table 2. The results based on peak absorption intensity can be summarized as follows: (1) The slope of the fitted line for the 5.58 THz peak is steeper than those for the 8.67 THz and 9.28 THz peaks, indicating that the 5.58 THz peak offers the highest sensitivity for warfarin quantification. (2) The R^2^ values for all fitted lines is > 0.998, indicating a strong correlation between the fitted and experimental data and demonstrating high reliability in quantitative analysis.

Furthermore, the linear relationship between the peak-integrated area and warfarin mass is depicted in Figure 5c, where we also constrained the intercept of the fitting function to zero for improved accuracy. The corresponding linear fitting function, which models the mass (*x*, in mg) versus peak area (*y*, in arbitrary units), is shown in Table 3. The key findings are as follows: (1) The slopes for the characteristic peaks at 8.67 THz and 9.28 THz are similar and lower than that at 5.58 THz, indicating that the highest sensitivity is at 5.58 THz. (2) The R^2^ values for all linear fitting results exceed 0.998, confirming a strong correlation between the fitted and experimental peak area and demonstrating high reliability of the fitting results.

To assess the efficacy of this dual-parametric quantification method, the limit of detection (LOD) was calculated using the following formula, which is based on the standard deviation and the slope [38]:

(1) $$LOD=\frac{3.3\delta }{S}$$

where *δ* is the standard deviation of the blank sample (see Table 4), and *S* is the slope of the calibration curve. The coefficient 3.3 is based on statistical principles, ensuring a 99% confidence level in distinguishing the signal from background noise [39,40].

The results reveal indicator-dependent sensitivity variations in THz quantification. For instance, the 8.67 THz peak demonstrates enhanced lowest detection limit through peak intensity (LOD = 0.702 mg) versus highest detection limit through peak intensity (LOD = 0.801 mg), attributable to differential parameter responsivity. Peak intensity sensitivity scales with oscillator strength modulation by warfarin mass, while the peak area sensitivity depends on spectral baseline stability and peak broadening effects. Through the synergistic evaluation of three diagnostic peaks (4.36, 5.58, and 8.67 THz), we achieved optimized detection performance with a composite LOD of 0.641 mg (Table 5), representing 23% improvement over single-indicator approaches (5.58 THz peak intensity: LOD = 0.835 mg). This dual-parametric quantification approach employed complementary information from peak intensity and area measurements across distinct vibrational modes, establishing a robust framework for pharmaceutical THz spectroscopy that transcends traditional single-peak analysis limitations.

## 3. Materials and Methods

### 3.1. Sample Preparation

Warfarin powder (purity ≥ 98%, CAS: 81-81-2) was purchased from Aladdin Biochemical Technology Co. (Shanghai, China). Cycloolefin copolymer powder (COC, particle size < 60 μm) was purchased from the Shanghai Institute of Nuclear Research (Shanghai, China). All materials were used without pretreatment.

For qualitative analysis, 4 mg of warfarin powder was precisely weighed using a microbalance (MS105DU/A, METTLER TOLEDO, Zurich, Switzerland, ±0.01 mg accuracy). The sample was subsequently compressed under 10 MPa to form a smooth tablet with a diameter of 4 mm and a thickness of 0.05 mm. It was sealed in an airtight bag containing desiccants (CAS: 112926-00-8) to maintain dryness and prevent moisture absorption. The sealed bag was then stored in a 4 °C refrigerator.

For quantitative analysis, to simulate commercial formulations (1 mg, 2.5 mg, 3 mg, and 5 mg), warfarin–COC mixtures (1–10 mg warfarin + 60 mg COC) were homogenized mixed and compressed under identical conditions (10 MPa) into 13 mm diameter tablets with thickness 0.8 mm. As detailed in Table 5, the mass loss during compression remained below 3.6%. All mixed samples were stored under the same conditions as those used for qualitative analysis.

### 3.2. Theoretical Calculation

The warfarin molecular structure was retrieved from the PubChem database for computational modeling (CID: 54678486). Quantum chemical calculations were performed using Gaussian 09W software package [41] with DFT method. For individual warfarin molecule, structural optimization and frequency analysis were carried out using the B3LYP/6-311G(d,p) basis set with GD3 dispersion correction [42,43]. For crystalline phase calculations, the unit cell structure was initially predicted using the Polymorph Predictor module within the Materials Studio 2020 package [44]. Subsequently, structural optimization and frequency analysis were conducted using the Gaussian 09W software package based on DFT, employing the B3LYP/6-31G(d,p) basis set with GD3 dispersion correction. Absorption spectrum analysis and vibrational mode visualization were performed using GaussView [45].

### 3.3. THz Spectroscopy Detection

THz absorption spectra were acquired using a commercial Fourier transform infrared spectrometer (FTIR, Vertex 80v, Bruker Optics, Ettlingen, Germany) equipped with DLaTGS/polyethylene detector. The signal-to-noise ratio of this equipment is 55,000:1. All spectra were measured with a spectral resolution of 4 cm^−1^, a scan number of 32, and a scan speed of 5 kHz. To minimize water vapor interference, all measurements were conducted in a vacuum environment (<300 pa) at room temperature (~22 °C).

Qualitative analysis employed pure warfarin pellets (4 mg), while quantitative analysis used a series of warfarin–COC mixed tablets. Warfarin was qualitatively detected by identifying its characteristic peaks using THz spectroscopy, while quantitative analysis was performed by examining the variation trends of peak intensity and peak area corresponding to different warfarin masses. To ensure data reliability, each sample was analyzed 3 times, and the data average was used for subsequent analysis.

## 4. Conclusions

This study establishes a dual-parametric THz spectroscopic approach for the comprehensive analysis of the widely used anticoagulant warfarin. Six characteristic absorption peaks at 4.36, 5.58, 6.73, 7.60, 8.67, and 9.28 THz were identified for warfarin between 4 and 10 THz. Four characteristic peaks (4.36, 5.58, 7.60, 9.28 THz) demonstrated excellent agreement (Δ < 0.16 THz) with single-molecule vibrational modes. The remaining peaks at 6.73 and 8.67 THz were resolved through crystalline lattice modeling, reducing deviations to <0.07 THz. Quantitative analysis revealed linear correlations (R^2^ > 0.998) between spectral parameters (peak intensity/area) and mass, achieving a 0.641 mg LOD that satisfies commercial tablet specifications. The results indicate that both the intensity and area of the characteristic peaks exhibit a linear increasing trend with the increasing warfarin mass (R^2^ exceeds 0.998), with an LOD of 0.641 mg, which meets or exceeds the minimum content requirements for commercially available warfarin tablets. The detection process requires less than 5 min per sample (vacuuming process: ~3 min; spectral data acquisition process: ~2 min) without chemical pre-treatment, demonstrating a significant time reduction compared to conventional HPLC methods. This work advances pharmaceutical analysis by enabling simultaneous molecular fingerprint identification and dosage quantification through non-destructive and rapid THz spectroscopy technology.

## Figures and Tables

**Table 1:** Correspondence between theoretical and measured warfarin peaks and the corresponding molecular vibrational patterns.

Theoretical Frequency/THz	Measured Frequency/THz	Deviation/THz	Molecular Motion
4.52	4.36	0.16	out-of-plane bending (C_9_H_5_O_3_)
5.74	5.58	0.16	rocking (C_9_H_5_O_3_) + rocking (CH_2_)
7.74	7.60	0.14	rocking (CH_2_) + rocking (Ph)
9.33	9.28	0.05	in-plane bending (C_9_H_5_O_3_)

**Table 2:** Linear fitting formulas and correlation coefficients for peak intensity versus warfarin mass.

Frequency/THz	Linear Fitting Function Expression	Correlation Coefficient/R^2^
5.58	*y* = 36.33*x*	0.998
8.67	*y* = 26.35*x*	0.999
9.28	*y* = 19.83*x*	0.999

**Table 3:** Linear fitting formulas and correlation coefficients for peak area versus warfarin mass.

Frequency/THz	Linear Fitting Function Expression	Correlation Coefficient/R^2^
5.58	*y* = 13.17 *x*	0.999
8.67	*y* = 5.27 *x*	0.998
9.28	*y* = 5.23 *x*	0.999

**Table 4:** Quantification performance of warfarin using a dual-parametric THz spectroscopic approach.

Frequency/THz	Peak Intensity/arb.u.			Peak Area/arb.u.		
	δ	S/(mg^−1^)	LOD/mg	δ	S/(mg^−1^)	LOD/mg
5.58	0.0092	0.03633	0.836	0.00256	0.01317	0.641
8.67	0.00561	0.02635	0.703	0.00128	0.00527	0.802
9.28	0.00425	0.01983	0.707	0.00123	0.00523	0.776

**Table 5:** Proportioning of samples for quantitative experiments.

Number	Warfarin/mg	COC/mg	Mass (Before Pressing)/mg	Mass (After Pressing)/mg	Mass Loss
1	1.18	60.43	61.61	59.37	3.6%
2	2.17	60.45	62.62	64.40	2.8%
3	3.19	60.77	63.96	62.20	2.8%
4	4.08	60.75	64.83	62.84	3.1%
5	5.12	60.75	65.87	64.29	2.4%
6	6.17	60.61	66.78	65.11	2.5%
7	7.12	60.63	67.75	65.72	3.0%
8	8.16	60.58	68.74	66.95	2.6%
9	9.17	60.55	69.72	67.48	3.2%
10	10.18	60.78	70.96	68.94	2.8%

## Data Availability

The original contributions presented in the study are included in the article; further inquiries can be directed to the corresponding authors.
